# Molecular Dynamics Study on the Deformation Behaviors of Nanostructures in the Demolding Process of Micro-Injection Molding

**DOI:** 10.3390/polym11030470

**Published:** 2019-03-12

**Authors:** Can Weng, Jin Yang, Dongjiao Yang, Bingyan Jiang

**Affiliations:** 1 College of Mechanical and Electrical Engineering, Central South University, Changsha 410083, China; canweng@csu.edu.cn (C.W.); yj20141116@csu.edu.cn (J.Y.); yangdongjiao@163.com (D.Y.); 2 State Key Laboratory of High Performance Complex Manufacturing, Central South University, Changsha 410083, China

**Keywords:** micro-injection molding, demolding process, molecular dynamics simulation, nanostructure, polymer

## Abstract

Polymer parts with nanostructures have broad applications, possessing excellent optical, electrochemical, biological, and other functions. Injection molding technology is one of the main methods for mass production of polymer parts with various shapes and sizes. The demolding process is vital to the replication quality of molded parts with nanostructures. For this study, molecular dynamics simulations of polypropylene (PP), polymethyl methacrylate (PMMA), and cycloolefin copolymer (COC) were conducted for the demolding process. The average velocity, density distribution, adhesion energy, and demolding resistance were introduced to analyze the deformation behaviors of polymer nanostructure from a nickel nano-cavity with an aspect ratio of 2:1. The shoulders of nanostructures were firstly separated from the nickel mold insert in the simulation. Under the external demolding force of 0.07 nN, PP and PMMA could be successfully demolded with some deformations, while COC could not be completely demolded due to the greater adhesion energy between COC and Ni. It was found that the maximum adhesion energy occurred in the separation process between the shoulder of the nanostructure and Ni and the huge adhesion energy was the main cause of demolding defects. The velocity difference of the whole polymer layer and polymer nanostructure was further analyzed to explain the nanostructure deformation. In order to improve the quality of demolding, the external force applied on polymers should be properly increased.

## 1. Introduction

With the progress of science and technology, especially the development of micro electro mechanical systems (MEMS), micro/nano fabrication technology has become a hot research topic [1]. It is a modern molding technology involving many disciplines, such as mechanics, materials, and microelectronics. The functional surface composed by micro/nano structures is a surface with regular texture, periodic arrangement, and micro/nano single scale or multi-scale features [2]. Thus, it could possess excellent optical, electrochemical, biological, and other functional characteristics [3,4,5,6]. High replication quality of nanostructures is critical for its proper function and the quality of molding directly affects the functions and characteristics of surfaces. The injection molding technology [7,8,9] is currently one of the main methods to fabricate polymer parts with various shapes and sizes. This micro/nano technology has many advantages, such as low cost, high efficiency, high precision, flexibility, and short processing cycle. It is gradually getting attention from many researchers.

During the micro-injection molding process, much quality damage to the parts with nanostructures may not occur in the filling and packing processes, but is caused by a coupling of factors in the demolding process, such as temperature change, uneven stress, and material property [10,11,12]. The interaction between molecules and atoms at the nanoscale cannot be neglected because of the scale effect. The van der Waals force at the interface between the polymers and the metal mold insert will increase sharply, when the surface structure or surface roughness decreases to the nanometer scale, even at a few tens of nanometers [13]. Therefore, it is interesting to study the mechanism of the interaction between molecules and the metal mold insert in the demolding process, which is helpful to guide the lossless replication of nanostructures. In the demolding stage, the polymer is mainly subjected to the friction force [14], the adhesion force [15], and the thermal contraction force [16] caused by different heat shrinkage coefficients of polymers and metal mold inserts. Fracture, elongation, necking, surface burr, and structural bending of nanostructures occur consequently [17,18]. The force acting on the workpiece not only brings its deformation but also influences its mechanical properties. Therefore, it is essential to analyze the force acting on a nanostructure as the polymer separates from the metal mold insert in the demolding process.

As the force components acting on nanostructure during the demolding process, Guo et al. [19] believed that the surface forces were generated by the combination of adhesion and friction force. Su et al. [20] found that the adhesion force had a bigger influence than the friction force when the processing parameters were optimized to minimize the demolding force during the demolding process. Sasak et al. [21] studied the demolding force for different polymer materials with different surface roughness molds. It was found that the demolding force was decreased as the surface roughness decreased and the situation was reverse when the mold surface roughness was lower than 0.2 μm. The demolding force in this case was mainly composed of the meniscus force and van der Waals forces. It can be seen that the surface and interfacial effects play a dominant role in the micro-injection molding process for nanostructures. The in-depth study of interface behaviors is thus needed to guide the demolding process.

Delaney et al. [22] summarized the existing demolding force models and parameters affecting the demolding force for micro polymer replication and pointed out that the existing models were not suitable for explaining the complex phenomena occurring at the interface between mold and polymer. After decades of development, the theory of molecular dynamics (MD) has been gradually applied to nanoscale research in many fields, such as macromolecular motion, nano-fluidic transport [23], interface science, and polymer forming processes [24]. Yang et al. [25] studied the influences of mold and substrate material combination on the adhesion characteristics in a thermal nanoimprint lithography (NIL) process by MD simulation. Takai et al. [26] established the MD model of the demolding process in NIL and analyzed the differences between the two simulation methods of MD and continuum mechanics. Kang et al. [27] used the MD simulation to study the changes of adhesion and friction force with different aspect ratios of nanostructures in NIL. For the MD simulation of the injection molding process at the nanoscale, only the filling stage has been explored by scholars [28,29] and research on the interface behavior between the polymer and the metal mold insert during the demolding process has not been reported. In view of the similarities between nanoimprint and micro-injection molding, the MD simulation of interface behavior during the nanoimprint process can provide a theoretical basis and technical guidance for this study.

In this study, the MD method was used to simulate the demolding process of micro-injection molding for three polymer materials in a nano-cavity with an aspect ratio of 2:1. Polymer models of PP, PMMA, COC, and a nickel nano-cavity model were constructed to form the polymer-metal systems. For simulation work, the optimum filling state was regarded as the initial model for the demolding process. The deformation behavior of nanostructures was investigated by analyzing snapshots at different demolding stages. Meanwhile, the forming qualities and deformation phenomena of nanostructures were explained by the variation of velocity distribution, adhesion energy, demolding resistance, and density distribution of polymers during the demolding process. The forming qualities of nanostructures under different external demolding forces were also studied.

## 2. Materials and Methods

### 2.1. Model Construction

**Figure 1:**
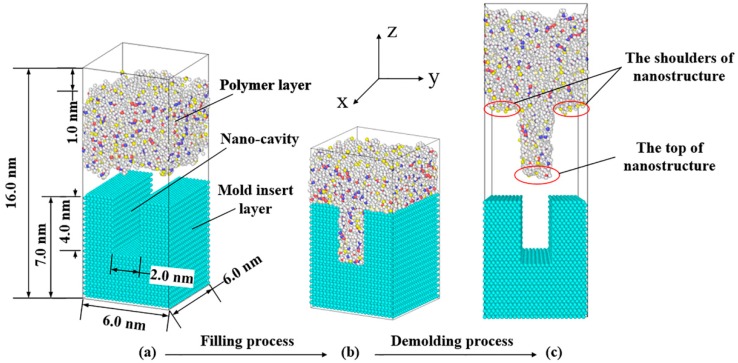
The molecular model of the micro-injection molding process.

The molecular models of the micro-injection molding process, composed of the upper polymer layer and the lower nickel mold insert layer with a nano-cavity, were established, as shown in Figure 1. PP, PMMA, and COC materials were chosen in this study. Taking COC as an example, the periodic COC amorphous system, with a box size of 60 × 60 × 38 Å, was established. The polymerization degree of each chain was 10 and the total number of chains was 100. The polymerization degree of COC used in real micro-injection molding was much larger than that considered in this study. In order to observe the separation of the nanostructure from the nano-cavity during the demolding process, a low polymerization degree was set to allow the molecular slippage and disentanglement under the external demolding force. The system temperature was 298 K and the initial density was 1.02 g/cm^3^. In order to remove the internal stress of the polymer system and make it balanced, the periodic polymer cell was optimized by the geometry optimization. Cycling annealing was performed under NVT condition with a high temperature of 550 K and a low temperature of 298 K. Finally, the COC system was heated up to 553 K to obtain a molten state. The constructions methods of the PP, PMMA, and COC models were similar to each other. In order to ensure that the model size and the atomic number of the three polymers were comparable to each other, the numbers of molecular chains of PP and PMMA models were 200 and 100, respectively. Their corresponding densities were 0.9 g/cm^3^ and 1.18 g/cm^3^. The annealing temperatures for PP and PMMA were both 500 K.

The mold insert was constructed by the unit cell of Ni as an FCC structure with a (1 0 0) plane. The height of Ni unit cell was 7.0 nm. The unit cell was expanded to establish a supercell structure of Ni. A nano-cavity with an aspect ratio of 2:1 was built in the Ni layer. The depth and width were 4.0 nm and 2.0 nm, respectively. Ni metal is easily oxidized in the air. In order to simplify the model building process, the oxide layer was not considered on the surface of the nickel mold insert. Since periodic boundary conditions were used in molecular systems, a vacuum layer with a thickness of 1.0 nm was added over the polymer layer.

### 2.2. Force Field

In this study, the polymer consistent force field (PCFF) in the consistent force field (CFF) of the second generation was adopted. Therefore, it can be used to calculate the self-action of molecules and their interaction with the atoms of Ni in the process of micro-injection molding. Both the energy minimization process and the molecular dynamics equilibrium process used the PCFF. The van der Waals and Coulomb were selected for the non-bond interaction, with a cut off distance of 1.25 nm.

### 2.3. Adhesion Energy

The interaction energy of the interface between polymer and Ni is mainly composed of van der Waals and electrostatic interaction energy [30,31], which can be approximated as the adhesion energy of the interface. Therefore, the adhesion energy between polymer and Ni was calculated by Equation (1), as follows:

(1) $$E_{adhesion}=E_{interaction}=E_{total}-\left(E_{polymer}+E_{Ni}\right)$$

where $E_{total}$, $E_{polymer}$ and $E_{Ni}$ are the total energy of polymer-Ni system, the surface energy of the polymer without the metal mold insert of Ni, and the surface energy of the metal mold insert of Ni without polymer, respectively.

### 2.4. Simulation Procedure

The whole simulation process was implemented by LAMMPS, an open source MD package in a computer cluster. Periodic boundary condition was selected in the X and Y directions and free boundary condition was selected in the Z direction. The PCFF and NVT ensemble were selected for the whole simulation process. In order to improve the calculation accuracy and observe the motion of the nanostructure during the demolding process, the time step was set to 0.1 fs and the total running steps were 40,000 steps. In the filling process, the injection force exerted on every atom of the polymers was selected as 0.07 nN (equal to 1.0 kcal/mol·Å). Meanwhile, the package time was set to 1.0 ps to completely fill the nano-cavity in the packing process. The filling temperatures of PP, PMMA, and COC were 523 K, 533 K, and 553 K, respectively. Then, the optimum filling state was regarded as the initial model of demolding. The demolding process began when the polymer temperature was dropped to 353 K. The external demolding force of 0.07 nN was also exerted on every atom of the polymers.

## 3. Results and Discussions

### 3.1. Simulation of Demolding Process

**Figure 2:**
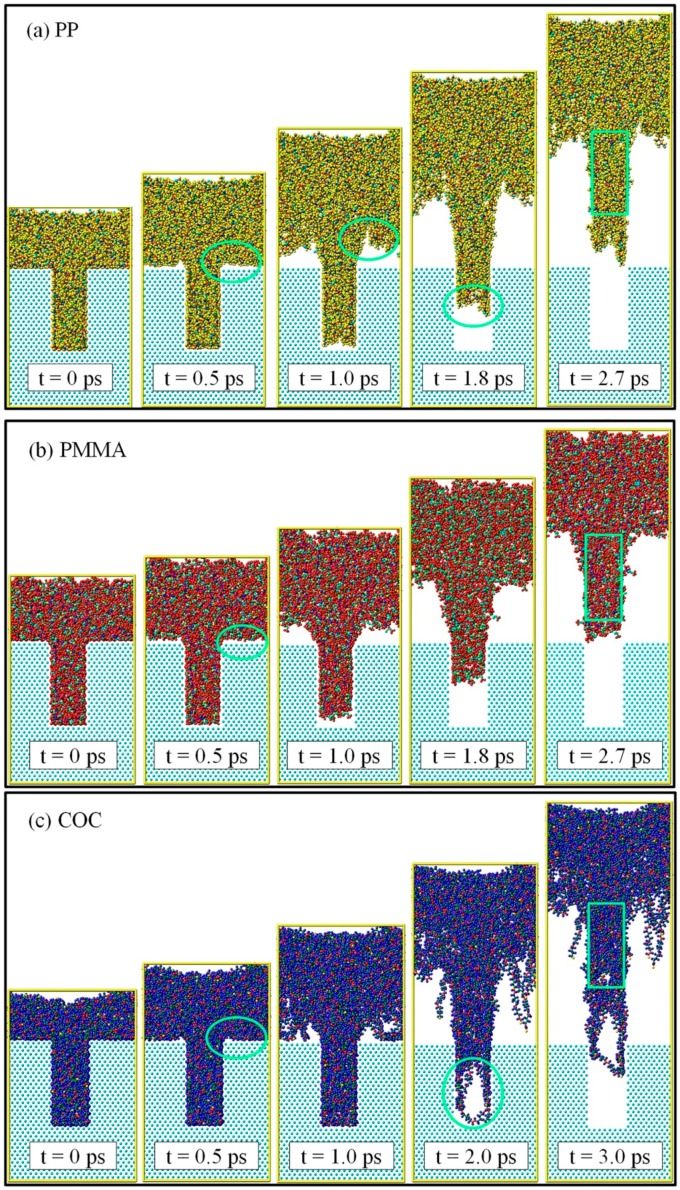
Snapshots of MD simulations of demolding processes for PP, PMMA, and COC.

The demolding processes of the three polymers are shown in Figure 2. The nano-cavities were fully filled by polymers at the time of 0 ps. It can be seen that PP and PMMA were successfully demolded, under the external demolding force of 0.07 nN, from the nano-cavities with the aspect ratio of 2:1. However, COC could not be completely demolded and had a seriously deformed nanostructure. Prior to the time of 0.5 ps, there was no obvious separation between polymers and Ni. However, the polymer molecules at the substrate began to move upward and the molecular chains were constantly pulling each other. As shown by the circles in Figure 2, the shoulders of nanostructures were observed to separate from the interface firstly. The nanostructures of PP and PMMA began to separate quickly from the nano-cavities at 1.0 ps. At this time, the shoulders of the PP nanostructure became uneven and presented necking deformation. Then, the necking deformation gradually increased with time until 1.8 ps. Consequently, the non-bonded interaction between the polymer and Ni during the demolding process might not end immediately with the separation of interface, due to the time dependence of molecular motion. PP and PMMA were completely separated from the nano-cavities almost simultaneously at 2.7 ps, but COC was not completely separated even at 3.0 ps. There was a serious depressed deformation at the top of the PP nanostructure and slight surface burr on the PMMA nanostructure. As for the COC, the nanostructure was significantly elongated and had many voids in it.

### 3.2. Velocity Distributions of Polymers

**Figure 3:**
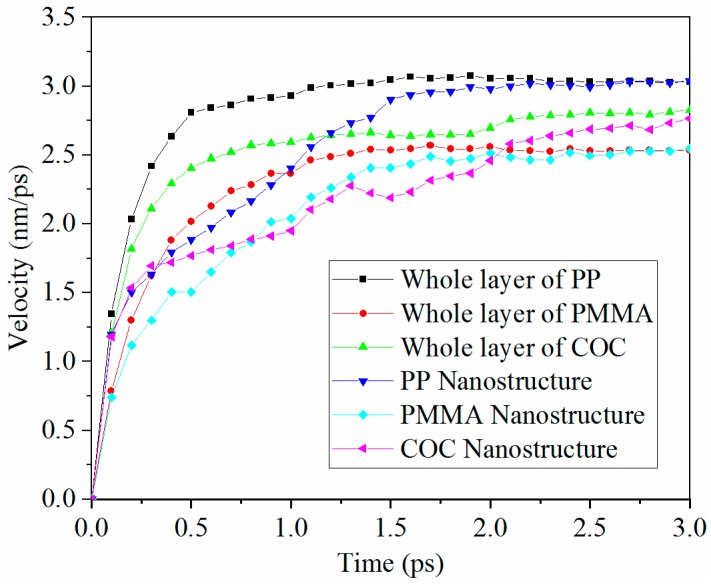
Average velocities of the whole layer and its nanostructure for each polymer during the demolding process.

The average velocity of the whole layer and its nanostructure for each polymer is illustrated in Figure 3. In the early stage of the demolding process, the velocities of the three polymers were relatively low, especially the velocity of nanostructure for each polymer, which was only about two-thirds of the whole polymer layer. The reason may be that polymer molecules in the nano-cavity area need to overcome the adhesion force between the polymers and the nickel surface. After overcoming the adhesion force, the polymers began to move out of the nano-cavity and the velocity was then greatly increased. Additionally, due to the friction force, the polymer molecules in the nano-cavity moved slower than the whole polymer layer. When the polymer was completely separated from the nano-cavity the velocity of the nanostructure was consistent with the whole polymer layer.

The reason for the elongated nanostructure was possibly the velocity difference between the whole polymer layer and the polymer nanostructure. Since its velocity difference was the smallest among three polymers, the elongation of the PMMA nanostructure was the least. The PP had the highest average velocity; thus, it was the first to complete the demolding process. The velocity of COC was larger than that of the PMMA, although the COC nanostructure was the most difficult one to be demolded. The external force applied to the COC failed to overcome the adhesion force with the mold insert, but it was greater than the interaction force of the molecular chains. As a result, the molecular chains in the middle of the COC nanostructure were separated from the molecular chains at the edge and then a higher velocity was obtained.

**Figure 4:**
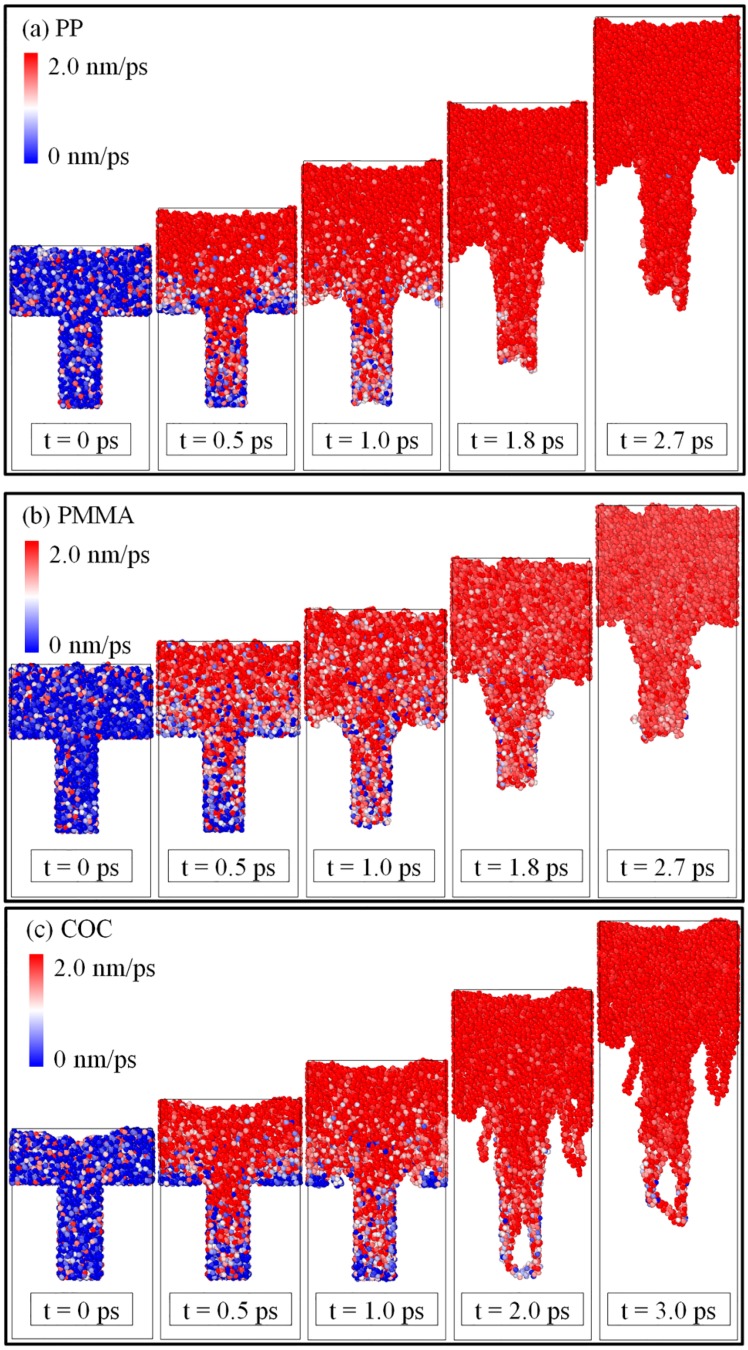
Distributions of atomic velocity of three polymers with time in the demolding process.

The velocity distributions of all atoms in the polymer layers, which are used to further explore the causes of nanostructure deformation, are shown in Figure 4. In the early stage of demolding, the velocities of the atoms at the shoulders and tops of the nanostructures were the slowest, due to the adhesion force with the mold insert. This may explain why the necking deformation of polymers occurred. The depression deformations at the tops of the nanostructures may be caused by the different velocities of atoms on the edge and on the inside. The lower velocity on the edge was caused by the adhesion force and the friction force at the interface. As in Figure 4b,c, it was found that the atomic velocity distribution of COC had a large difference compared to PMMA in the late demolding process. The atomic velocities of PMMA were generally lower than COC due to its dense structure. This may also explain why the velocity of COC was larger than that of PMMA.

### 3.3. Adhesion Energies and Demolding Resistances of Polymers

**Figure 5:**
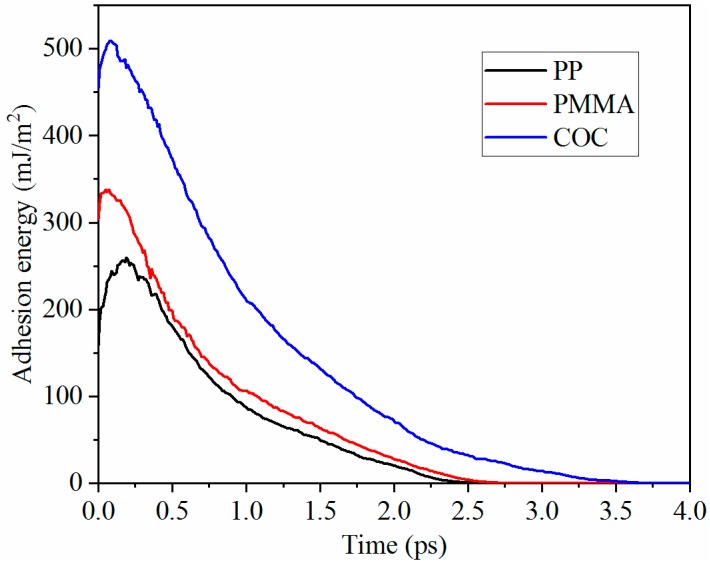
Adhesion energies at the interface between each polymer and Ni.

The adhesion energy was further analyzed to explore the demolding process for each polymer. The adhesion energies at the interfaces between polymers and Ni are shown in Figure 5. The adhesion energy of each polymer first increased and then drastically decreased with time and finally approached zero when the nanostructure was completely demolded from the nano-cavity. This showed that the non-bonded interaction at the interface did not exist. Considering the demolding process, the maximum adhesion energy might occur in the separation process between the shoulders of the nanostructure and the Ni. The peak values of adhesion energy for PP, PMMA, and COC were 259.57 mJ/m^2^, 338.32 mJ/m^2^, and 509.29 mJ/m^2^, respectively. The adhesion energy for COC was much larger than the other two polymers. Therefore, PP and PMMA nanostructures could be completely demolded with slight deformation. The large adhesion energy may bring demolding difficulty for the COC material and the dispersion and hysteresis of COC molecular chains.

**Figure 6:**
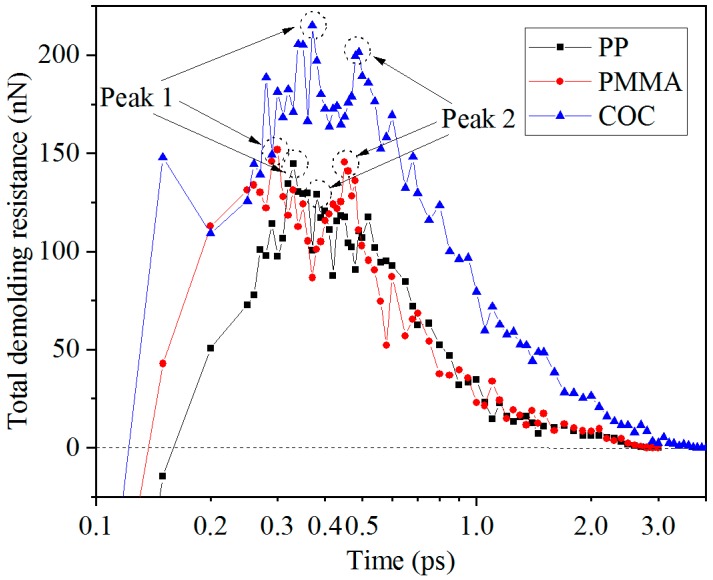
Total demolding resistances of the three polymers.

Polymer atoms are mainly subjected to the applied external force, the adhesion force, the thermal contraction force, and the friction force during the demolding process. The total demolding resistance for polymers was the sum of every atomic resistance in Z direction, as shown in Figure 6. It can be seen that the total demolding resistances were increased sharply and then gradually decreased to zero. There were two peaks in the curves between 0.3 ps and 0.5 ps. The two peaks may be caused by overcoming the adhesion force when the shoulders and the top of nanostructures were separated from the Ni surface, respectively. The first peak was slightly larger than the second one, perhaps because the adhesion energy between the shoulder of the nanostructure and the Ni was larger than that between the top of the nanostructure and the Ni.

The adhesion force, which made the molecular chains in the nanostructure stretch, was the main source of deformation force at the initial stage of demolding. Then, the friction force played a role in subjecting the atoms on the edges of the nanostructures to a larger force with a slower speed. Thus, the nanostructures would elongate and thicken. The total demolding resistance of COC was much greater than the other two polymers. It was difficult for COC to achieve complete demolding, which resulted in the molecular chain’s dispersion and hysteresis. The total demolding resistance of PMMA was larger than that of PP in the early stage of demolding and then the difference between them became small in the middle and later stages. The reason for this phenomenon may be that the adhesion energy of PMMA was much larger than that of PP at the initial stage of demolding and then the difference of adhesion energy was gradually reduced in the middle and later stages.

### 3.4. Density Distributions of Polymers

**Figure 7:**
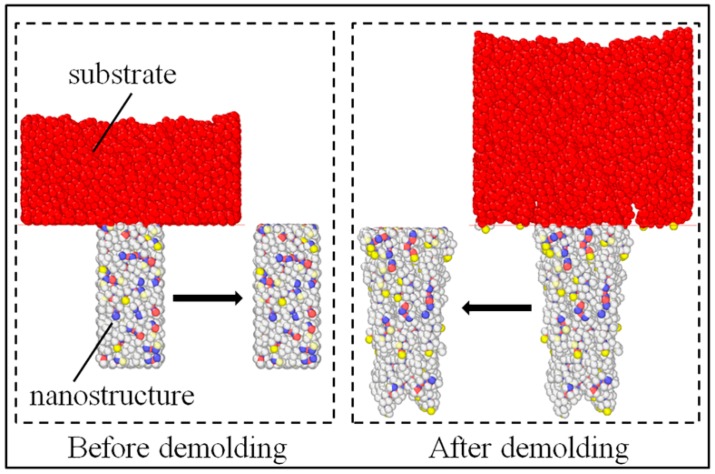
Schematic diagram of partitioning polymer nanostructures.

**Figure 8:**
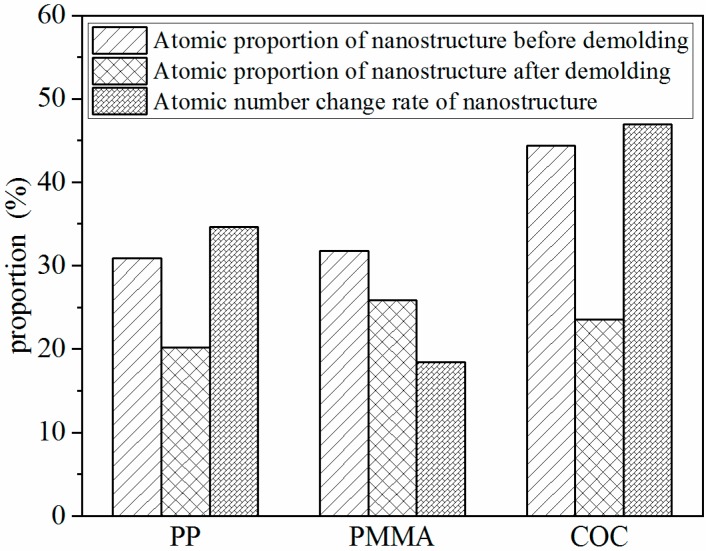
Density variations of nanostructures of each polymer.

The numbers of atoms in the nanostructure and the whole layer at the initial and final time of the demolding process were calculated to represent the density changes of the nanostructure before and after demolding. The polymer nanostructures, as shown in Figure 7, were partitioned from the whole layer. The atomic proportion of the nanostructure was calculated by Equation (2), as follows:

(2) $$K_{ap}\left(\%\right)=\frac{N_{n}}{N_{w}}\times 100\%$$

where $N_{n}$ and $N_{w}$ are the total numbers of atoms in the nanostructure and the whole layer, respectively. The atomic number change rate of the nanostructure was calculated by Equation (3), as follows:

(3) $$K_{cr}\left(\%\right)=\frac{N_{r}}{N_{w}}\times 100\%$$

where $N_{r}$ and $N_{w}$ are the number of atomic reductions of nanostructure after demolding and the total number of atoms in the whole layer, respectively. As can be seen in Figure 8, the densities of the three polymers obviously decreased. As the three polymers were subjected to the adhesion force and friction force during the demolding process, the dense molecular chains became loose after demolding. With the same aspect ratio, the filling rate of COC was the highest, due to the good flowability. Consequently, the density of the COC nanostructure was the largest after the filling stage. However, owing to the large adhesion energy at the interface between COC and Ni, the density of the nanostructure was changed the most.

**Figure 9:**
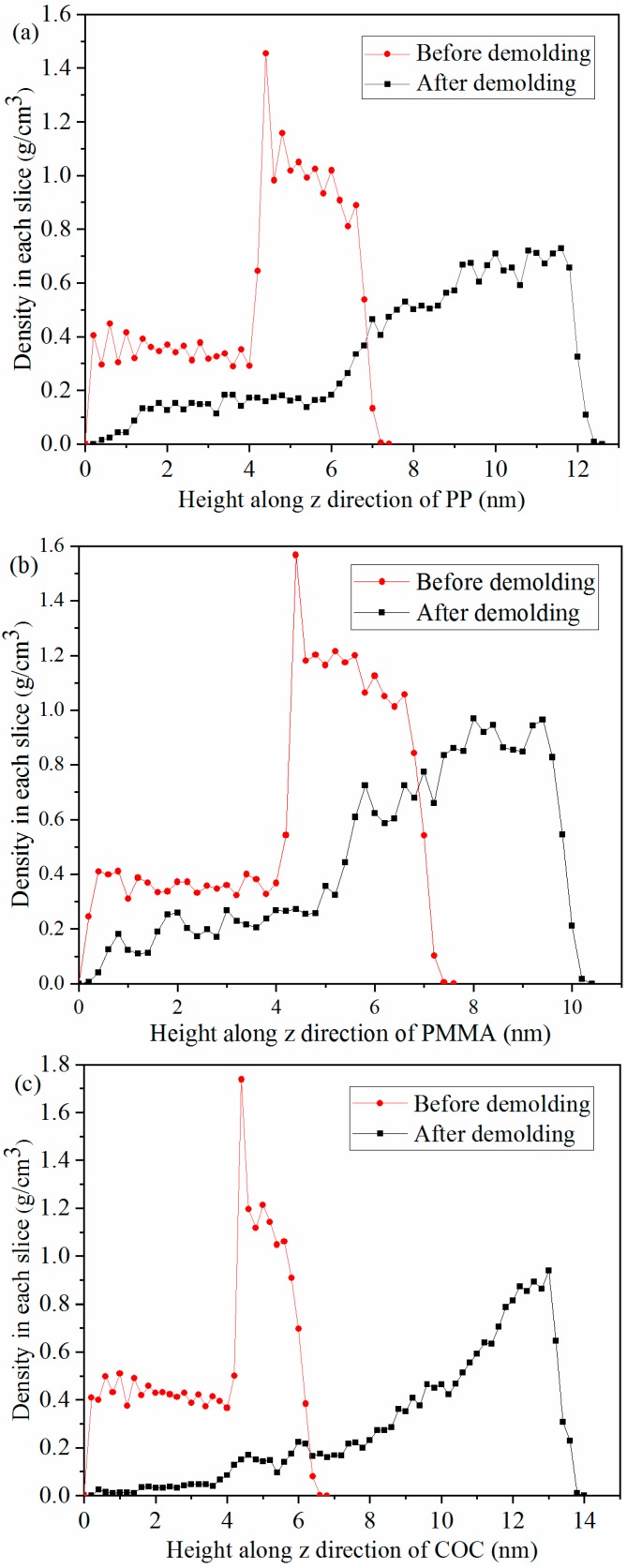
Density in each slice of the three polymers before and after demolding.

Additionally, the cohesive energy density (CED), scaling the magnitude of the intermolecular force, was calculated after filling completely, to explain the density variation. The CED of the three polymers was calculated by Equation (4), as follows:

(4) $$CED=\frac{E_{coh}}{V_{m}}$$

where $E_{coh}$ and $V_{m}$ are the total cohesive energy and the molar volume of the polymer, respectively [32]. The total cohesion energies of the three polymers were calculated by LAMMPS, and the values of CED of PP, PMMA, and COC were 248.41 MJ/m^3^, 329.34 MJ/m^3^, and 258.02 MJ/m^3^, respectively. The results showed that the CED of PMMA was the largest, followed by COC, and PP was the smallest. This may be due to the fact that there were no polar groups in the molecular chains of PP and COC and the main intermolecular force was the dispersion force. However, there were polar groups in the molecular chains of PMMA and the intermolecular force was mainly the dipole–dipole force. Thus, the interaction between PMMA molecules was stronger than that of PP and COC. The number of atoms that decreased in the nanostructures during demolding was the greatest for COC, second for PP, and lowest for PMMA. The lowest, for PMMA, may be attributed to the largest CED. Since the CED of PP was smaller than that of PMMA, the density change of PP was larger than that of PMMA. Figure 9 demonstrates the density distributions of polymers in each slice along the Z direction, with a slice thickness of 0.2 nm. It can be seen that the density of the whole polymer layer decreased after demolding and that the nanostructures became obviously longer. Prior to demolding, the layer density at the shoulder of nanostructures was changed abruptly and reached a maximum. The maximum values of PP, PMMA, and COC were 1.45 g/cm^3^, 1.56 g/cm^3^, and 1.73 g/cm^3^, respectively. Therefore, the corresponded maximum densities of the three polymers appeared on the shoulders of the nanostructures after the filling and packing stages.

### 3.5. Effects of External Demolding Force

**Figure 10:**
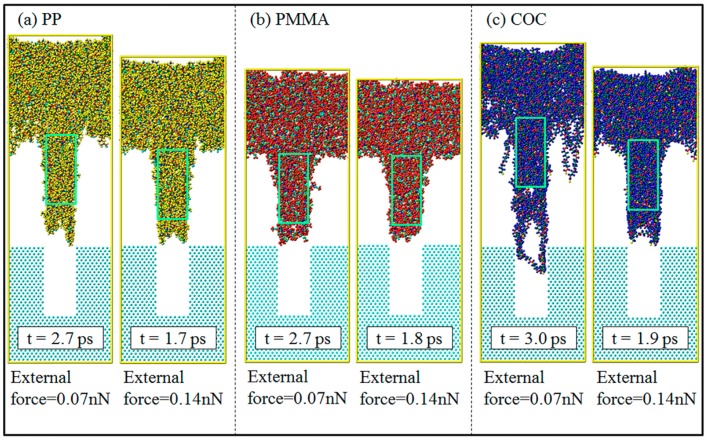
Snapshots of results for completed demolding under different external forces.

Under the external force of 0.07 nN, PP and PMMA could be successfully demolded, with some deformations and defects, but COC could not be demolded completely. The external force was then increased to 0.14 nN (equal to 2.0 kcal/mol·Å). The snapshots of results for completed demolding, under different external forces, are shown in Figure 10. When the external force was 0.14 nN, the three polymers could be all smoothly demolded and the replication qualities of nanostructures were better than those with the external force of 0.07 nN. After increasing the external force, the defects of elongation and surface burr on the nanostructures were reduced. The COC could overcome the adhesion force with non-stretched molecular chains. When the demolding was completed, the top of nanostructures possessed the similar depressed deformation of PP but the overall structure basically kept its original appearance. In addition, the demolding time was greatly shortened with a larger external force. The PP took only 1.7 ps to complete the demolding and the COC also shortened the demolding time to 1.9 ps.

**Figure 11:**
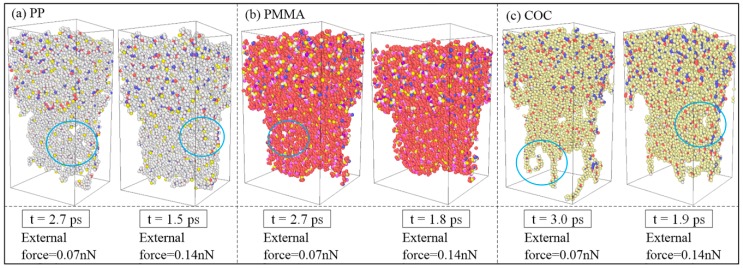
Three-dimensional topographies of results for completed demolding under different external forces.

In order to clearly observe the internal defects of nanostructures, three-dimensional topographies were derived after demolding, as shown in Figure 11. When the external force was 0.07 nN, the nanostructures presented different internal defects. The COC nanostructure was the most serious one, with many voids. The structural voids of PP and PMMA mainly occurred inside the nanostructure, while voids mainly appeared on the edges of COC nanostructure. This was also due to the greater adhesion energy between COC and Ni. With the increase of external force, the internal voids of nanostructures were obviously reduced. The replication qualities of the nanostructures were greatly improved. Properly increasing the external force of demolding would therefore be helpful to achieve the goal of non-destructive demolding for nanostructures.

## 4. Conclusions

In the present work, molecular dynamics simulations were conducted to investigate the deformation behaviors of nanostructures for three polymers in the demolding process of micro-injection molding. Under the external demolding force of 0.07 nN, PP, and PMMA nanostructures were completely demolded, with slight elongation, necking, and surface burr deformation, from nickel nano-cavities with an aspect ratio of 2:1. The COC nanostructure was not successfully demolded and had many serious defects, due to the larger adhesion energy between COC and Ni. The elongation deformation of nanostructures was caused by the inconsistent velocities of the whole polymer layer and polymer nanostructure. Similarly, the depression deformation at the top of the nanostructure was caused by the different velocities of atoms at the edge and inside of it. The peak values of adhesion energy occurred in the separation process between the shoulders of the nanostructure and the Ni. The peak values for PP, PMMA, and COC were 259.57 mJ/m^2^, 338.32 mJ/m^2^, and 509.29 mJ/m^2^, respectively. The total demolding resistance of COC was much greater than the other two polymers, which caused dispersion and hysteresis in the molecular chains of the nanostructure. After demolding, the densities of the three polymers were all decreased and the PMMA changed least due to the largest CED between its molecules. When the external demolding force increased to 0.14 nN, the demolding qualities of nanostructures were greatly improved. Therefore, appropriately increasing the external force applied on polymers is helpful to the non-destructive demolding of nanostructures.
